# Biomaterials for Cell Manufacturing

**DOI:** 10.1021/acsmacrolett.4c00634

**Published:** 2024-10-28

**Authors:** Ryan C. Miller, Johnna S. Temenoff

**Affiliations:** † Wallace H. Coulter Department of Biomedical Engineering, Georgia Tech/Emory University, Atlanta, Georgia 30332, United States; ‡ Parker H. Petit Institute for Bioengineering and Bioscience, Georgia Institute of Technology, Atlanta, Georgia 30332, United States

## Abstract

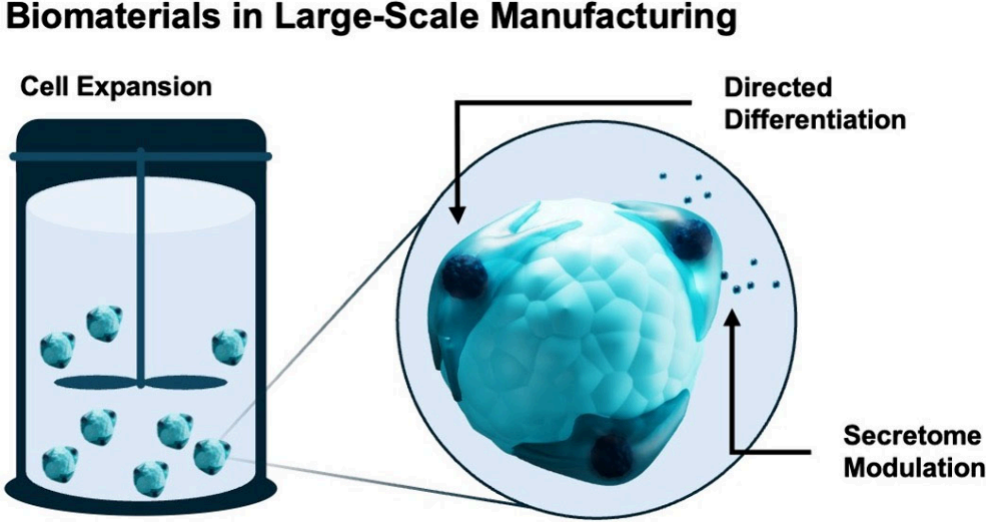

Cell therapies, potent populations of cells used to treat disease and injury, can be strategically manufactured with biomaterial intervention to improve clinical translation. In this viewpoint, we discuss biomaterial design and integration into cell manufacturing steps to achieve three main goals: scale-up, phenotype control, and selection of potent cells. Material properties can be engineered to influence the cell–biomaterial interface and, therefore, impart desirable cell behavior such as growth, secretory activity, and differentiation. Future directions for the field should capitalize on the combinatorial design of biomaterial properties to yield highly specific and potent cell populations. Furthermore, future biomaterials could contribute to novel high-throughput cell separation technologies that can individually select the most therapeutically relevant cells within a produced batch.

## 1 Introduction

### 1.1 Cell Therapies in the Clinic

Cell therapies, viable and dynamic cell populations that can be transferred into the body to prevent or treat disease, are of high interest to the research and clinical community following the USFDA approval of the first products in 2010.^1,2^ Unlike small molecule pharmaceutics, cell therapies capitalize on sophisticated mechanisms of action that can influence and integrate with multiple simultaneous healing processes. With this potential and due to the convergence of biomaterials, stem cell biology, immunotherapy, and gene editing research fields, there are currently thousands of active clinical trials for cell therapies.^3−5^ More than 10,000 open clinical trials, of which 214 are in phase IV, were identified following the search for “cell therapy” on the ClinicalTrials.gov database as of 2024. Although many clinical trials involving cell-based therapeutics have shown promising results, reproducible manufacturing of highly potent cells at scale remains a major limitation.^3,6−8^

### 1.2 Autologous and Allogenic Cell Therapies

Therapeutic cells can be derived from the patient or come from donor tissue. Autologous transplantation uses cells from the patient. As such, the likelihood for immune rejection is reduced,^3^ but often the cells must be culture expanded prior to reintroduction to the patient.^3^ Allogenic cells, or those that come from another person, benefit from the ability to store and/or bank the cells and therefore provide immediate availability for the treatment of many patients. As such, allogenic transplantation is better suited for urgent medical situations and has become a major research emphasis with regards to commercial-scale manufacturing.^3,9^

### 1.3 Scalable Cell Culture

Large-scale manufacturing of therapeutic cells remains a bottleneck to clinical translation and eventually commercialization.^4,6,9−11^ Therapeutic cell doses vary but are on the order of 10^9^ cells per patient.^6^ Due to these large dose requirements, conventional 2D culture flasks demonstrate limitations associated with large footprints and large process volumes at scale.^6,7^ As such, several 3D culture platforms have been adopted to address these shortcomings.

In this viewpoint, we will discuss bioreactor design for adherent cell culture systems since there is a direct interaction between cells and engineered biomaterial surfaces in this case. Several 3D hollow-fiber systems have been cleared by the FDA for clinical use.^7^ The use of microcarriers (microparticles of approximately 125–300 μm diameter^12^ used to increase surface area for cell attachment) suspended in a stir-tank reactor provide an alternative approach and can seamlessly incorporate therapeutic specific biomaterial design.^6,7^ To date, microparticle design has primarily focused on increasing cell proliferation rates to capitalize on yield with minimal emphasis on potency engineering.^4−6^ Nevertheless, biomaterials, as coatings in hollow-fiber reactors or as microcarriers, provide a great opportunity to synergize both phenotypic control and cell growth. However, the optimization of biomaterials for specific cell phenotypes remains a challenge.

### 1.4 Engineering Cell Potency

Therapeutic cell potency will be defined for the purposes of this viewpoint as the capacity of a cell to beneficially regulate the healing process. This can be achieved through enhanced paracrine signaling, differentiation, or tissue remodeling and therefore defines the ideal phenotypic state post manufacturing.^3,7,13,14^ The cell phenotype can be engineered genetically, usually by a viral vector, or directly through the use of transcription factors or biomaterials.^15,16^ More specifically, direct control of cell potency does not involve driving the cells to pass through a pluripotent stem cell stage.^15,17−19^ Genetic engineering of cell potency is a powerful regenerative medicine tool that can yield highly specific cell fates; however, it can lead to epigenetic remodeling and tumor formation.^15,20^ As such, genetic engineering will only be briefly discussed in this viewpoint. Direct engineering of cell potency, on the other hand, provides an alternative approach to cell fate control that has a low risk for epigenetic remodeling.^15^ While transcription factors were initially the standard for direct engineering of cell potency, the efficiency is low.^16^ Biomaterials, however, can provide physical and/or biochemical cues to increase the efficiency of direct engineering of cell potency, either through reprogramming or shifts in the phenotypic state.^15−18^ Although biomaterials have been used to engineer more theoretically potent cell populations, potency variability remains a translational challenge.

### 1.5 Heterogeneity in Cell Culture

Finally, in order to observe consistent therapeutic outcomes, variation in the cell phenotype should be minimized. Functional heterogeneity can exist within a single population of therapeutic cells or exist as batch-to-batch differences.^21−23^ Initial cell populations may vary in phenotype and efficacy based on parameters such as donor age, donor sex, donor condition, or cell source.^3^ For example, it is well-known that cellular senescence is highly correlated with age.^24^ Not only may cells from an elderly donor lead to a different therapeutic outcome compared to a young donor, but also the fraction of senescent cells within the donor population can have an entirely unique and deleterious secretome, called the senescent-associated secretory phenotype (SASP).^24,25^ While heterogeneity can partially be attributed to stochasticity, cells are also very sensitive to microenvironmental cues. As such, phenotypic changes can arise and compound during many manufacturing steps.^3,21,26^ Again, biomaterials provide an approach to reducing cell heterogeneity either through direct sorting/filtering of cell populations or indirectly through cell phenotype control. Selection of a subpopulation of cells at the end of the manufacturing process provides a methodology to reduce variability in therapeutic efficacy; however, many of these techniques have not been fully integrated into the manufacturing pipeline.

### 1.6 Viewpoint Goal

**Figure 1:**
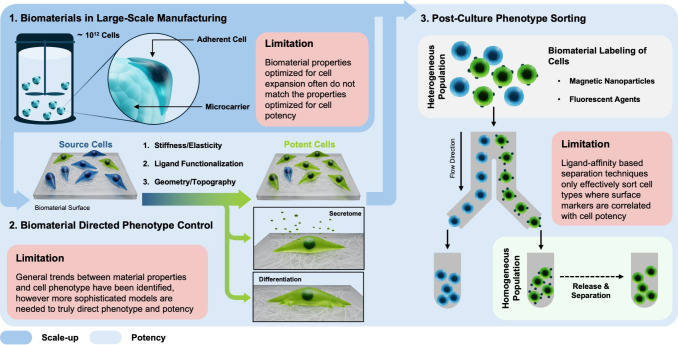
Biomaterial integration into the cell therapy manufacturing pipeline. Biomaterials can be used to both facilitate commercial scale-up of cells and impart cell potency to increase clinical efficacy. For adherent cell culture, cells can be expanded on microcarriers in suspension to reach clinically relevant lot sizes (10^12^ cells). The properties of the microcarrier (stiffness/elasticity, ligand functionalization, and geometry/topography) can be independently controlled to further facilitate cell growth and increase the potency of the source cells. The potency can take the form of directed differentiation of progenitor cells or modulation of the cell’s secretory phenotype. Following expansion and phenotype control, heterogeneity in the cell population can be reduced with various cell sorting technologies. Obtaining a homogeneous population of therapeutic cells will improve the reproducibility of potency outcomes.

This viewpoint will summarize current biomaterial strategies as well as highlight challenges that can be further addressed by biomaterials scientists in order to scale-up the manufacture of therapeutic cells and improve cell potency and specificity (Figure 1). In this viewpoint, advances in the culture of induced pluripotent stem cells (iPSCs) and mesenchymal stromal cells (MSCs) will be used as examples, but the general principles discussed can be extended to all clinically relevant cell types.

## 2 Biomaterials for Scalable Cell Culture – Increased Cell Yield

Clinical translation of cell therapies demands cost-effective scale-up of adherent cell manufacturing to achieve commercially relevant lot sizes.^6,7,11,27−29^ A single therapeutic dose is on the order of 10^9^ cells, and therefore, for multiple dose therapies and homogeneity of outcome, lot sizes should ideally be on the order of 10^12^ cells.^6,30^ Conventional planar technologies, although modified to contain multiple layers per vessel, have significant cost limitations at the scale needed to produce commercial-sized lots due to a low surface area to volume ratio (SA:V). As such, 3D systems, e.g., hollow-fiber and microcarrier suspension bioreactors, have been designed to maximize the SA:V (upward of a 100-fold increase) and therefore reduce both the footprint and cost. Furthermore, biomaterial design can be leveraged in 3D bioreactor systems to maximize the expansion rate through material properties.^11^ Of the various 3D systems, microcarrier-based suspension bioreactors provide a promising approach to capitalize on both optimized SA:V culture geometries and biomaterial intervention for improved growth kinetics.

### 2.1 Biomaterial-Based Microcarriers

As mentioned, microcarriers are used in suspension culture to increase the surface area for cell attachment, therefore maximizing cell growth during each passage. For many cell types, long-term expansion can lead to the development of adverse phenotypes such as increased cellular senescence or loss of multipotency.^31,32^ Therefore, maximizing the cell density is essential. Not only can microcarriers be engineered from different materials to optimize cell growth kinetics, but also microcarrier material properties can be used to enhance the effects of media additives.^11^ The minimization of expensive culture media additives provides another avenue for direct manufacturing cost reduction.^33,34^

#### 2.1.1 Nonporous and Macroporous Microcarriers

Broadly, microcarriers can be classified as either non- or macroporous. Several commercially available nonporous microcarriers exist such as Cytodex (cross-linked dextran matrix) and Synthemax (United States Patent Class VI polystyrene material).^27^ Mouse embryonic stem cells (ESCs) cultured on dextran microcarriers and human MSCs cultured on polystyrene carriers were shown to have a 200-fold and 1000-fold expansion, respectively, while maintaining their stemness.^35,36^

Compared to porous microcarriers, nonporous microcarriers allow easier cell seeding and harvesting; however, larger culture times are needed to achieve the same expansion due to limited cell–cell contact early after seeding.^27^ Like nonporous microcarriers, there are several commercially available macroporous microcarriers (e.g., CultiSpher-S). Mouse ESCs cultured on cross-linked gelatin microcarriers led to a 439-fold expansion in 6 days while maintaining pluripotency.^37^ Human MSCs cultured on gelatin-cross-linked microcarriers achieved a 1000-fold expansion, similar to Synthemax microcarriers; however, the 1000-fold expansion occurred 10 days faster.^38^ Macroporous microcarriers provide a larger surface area for cell adherence and migration than nonporous microcarriers. They also provide a structure that can protect against shear-induced phenotypic changes caused by impellers that can be used in suspension bioreactors.^27^ However, cell harvesting is more challenging and leads to a reduced cell yield. Therefore, while suspension bioreactor design can address the demands for commercial scale-up, the collection of cultured cells for downstream processing should be considered during process development.

#### 2.1.2 Degradable Microcarriers

For therapeutic approaches involving direct injection into a tissue defect, delivery of cells on microcarriers is a suitable option, and detachment of the cells prior to injection can be avoided. However, for cell therapies administered through alternative routes, cell detachment and isolation is necessary. Harvesting cells without inducing cell damage or phenotypic changes however remains a challenge.^27^ For example, traditional harvesting procedures utilize recombinant proteases to detach cells from their substrate that can lead to the damage of cell membrane proteins and receptors.^39^ As such, degradable microcarriers that are sensitive to pH, temperature, or enzymes have been engineered to better harvest cells.^40^ One such system using gelatin methacryloyl (GelMA) illustrated that a 5 min soft-digestion step (0.005% TrypZEAN, 10-fold lower than the recommended working concentration) could completely recover the human iPSC derived MSCs, maintain >95% viability, and preserve the immunomodulatory activity of the cells.^41^

### 2.2 Opportunities and Challenges

Although there has been progress in microcarrier design for the commercial scale-up of cell therapies, there is still an opportunity to exploit cell–biomaterial interactions to (1) optimize microcarrier formulations to enhance proliferation for specific cell types, (2) reduce costs associated with expensive media supplements, (3) increase cell expansion through the reduction of cellular senescence, and (4) increase the potency by artificially selecting desirable cell phenotypes. Furthermore, biomaterial advances mostly have been explored in the context of planar systems, but much of this work has not yet been translated to microcarrier systems.

Going forward, microcarrier research in the context of cell therapy manufacturing can capitalize on combinatorial biomaterial screening platforms to select for candidate microcarrier formulations.^42−46^ For example, hydrogel arrays using surfaces patterned through differential wettability have demonstrated the ability to probe both substrate stiffness and peptide immobilization on human MSC proliferation.^42^ It was found that 5 kPa polyethylene glycol (PEG)-based hydrogels with 4 mM CRGDS peptide led to the largest increase in human MSC proliferation.^42^ This same array setup was also used to screen for substrate properties that facilitate the activation of iPSC-derived vascular endothelial cells and illustrate the potential for a single platform technology to optimize microcarrier design for multiple different cells.^43^

Furthermore, similar technologies can screen for cell behavior other than proliferation, such as secretory activity and differentiation potential, which will be discussed later in this viewpoint. In an approach to reduce the serum content during the manufacture of MSCs, culture on heparin/collagen multilayers was found to support equivalent growth in 2% serum compared to conventional culture in 10% serum.^47^ Recently, layer-by-layer assembly of heparin/collagen surfaces was partially translated to 3D microcarrier systems.^48,49^ Similar to the planar study, it was demonstrated that the 2% serum condition supported equivalent growth to conventional culture in 10% serum.^48^ Similarly, polyethylene glycol coating of polystyrene microcarriers facilitated equivalent growth in serum-free media to growth on uncoated microcarriers in 10% FBS.^50,51^ Although promising, additional work is required to characterize the effects of surface coating of microcarriers on long-term expansion in a bioreactor.

Tuning the mechanical properties of hydrogel microcarriers was found to influence cellular senescence in MSCs.^25^ Serial culture on 100 kPa hydrogel surfaces reduced cellular senescence, characterized by β-galactosidase activity, compared to culture on conventional tissue culture plastic.^25^ Currently, only a few studies have begun to explore the MSC culture on hydrogel microcarriers as a mechanism to reduce cellular senescence. Again, translation of 2D hydrogel design into 3D systems presents a great opportunity to address the limitation of extended cell culture at scale.

Finally, hydrogel microcarrier design can be modulated to drive shifts in a cell’s secretory profile. For example, human MSCs, genetically modified to express the interleukin-1 receptor antagonist (IL-1Ra), cultured on microcarriers softer than commercial polystyrene microcarriers showed a marked increase in IL-1Ra secretion.^52^ While commercial microcarrier formulations are focused on proliferation, the biomaterials field can further improve microcarrier design by incorporating engineering principles aimed to modulate a cell’s phenotypic state, which will be emphasized in the next section.

## 3 Biomaterials for Controlling Cell Phenotypic Expression – Increased Cell Potency and Specificity

Cell–biomaterial interactions can be used to direct cells toward favorable functional states, increasing their specificity and therefore their potency.^3,5^ As mentioned in the Introduction, cell potency is unique to the disease and treatment mechanism and therefore can be controlled many ways such as through the modulation of the secretory behavior of the cells or by directed differentiation. Biomaterial control of both will be further discussed in this section.

Synthetic and natural biomaterials have a wide range of tunable properties that can be used to control cellular perturbations during manufacturing. Several studies have shown that cells are most similar to their in vivo phenotype when they are cultured on biomaterials resembling their native physiological niche.^53,54^ Therefore, cell niche engineering strategies have been largely motivated by our understanding of spatiotemporal and microenvironmental cues that direct cell behavior during wound healing and tissue remodeling.^53^ Matrix properties such as stiffness, shape, composition, and topography can be sensed by cells through cell surface receptors and therefore influence the cell state.^5,25,55^

### 3.1 Secretome Modulation

During healing, biological processes are often orchestrated by a complex interplay of multiple cytokines and growth factors and therefore would benefit from multimodal therapeutic intervention. Accordingly, exploiting the secretory activity of delivered stem cells has become a common therapeutic approach.^56−58^ The potency of the delivered stem cells can be enhanced through targeted shifts in their secretome profile using biomaterials.^4,5,58,59^ Mesenchymal stromal cells (MSCs) are of particular clinical interest due to their high secretory activity aiding in their immunomodulatory, angiogenic, and trophic properties.^5,25,60,61^ Their versatile secretion profiles have therefore been used to treat a diverse set of diseases and injury such as critical limb ischemia and Crohn’s disease.^61,62^

#### 3.1.1 Stiffness/Elasticity

With regards to human MSCs, mechanical cues of the substrate are known to yield unique secretory profiles.^25,56,61,63−65^ One potential mechanism for this observation is that increased matrix stiffness leading to increase cytoskeletal tension can facilitate the translocation of transcription regulators into the nucleus and initiate a cascade of downstream gene expression.^61^ In one example, MSCs cultured on polyethylene glycol diacrylate (PEGDA) hydrogels functionalized with integrin-engaging RGD peptides demonstrated a significant upregulation of a number of paracrine factors on 30 kPa gels compared to 100 kPa gels.^25^ Subsequent coculture of the secretome from MSCs cultured on 30 kPa gels with human umbilical cord endothelial cells initiated vessel network formation, demonstrating bioactivity of the secreted factors.^25^

In addition to the secretion of proregenerative factors, stiffness can be used to direct the immunomodulatory properties of MSCs. In one study, peptoids of different secondary structures were used as hydrogel cross-linkers to tune the stiffness.^66^ The softer (∼1.5 kPa) hydrogels resulted in increased immunoregulatory cytokine (e.g., IL-6, MCP-1, and M-CSF) secretion and gene expression.^66^ Similar results were reported for MSC spheroids encapsulated in 5 kPa alginate gels.^67^

In order to recapitulate all aspects of mechanical properties, viscoelastic gels have been synthesized using a combination of covalent and ionic linkers. For example, only cultures of MSCs on low stiffness (∼1 kPa) and energy dissipating (tan δ > 1) substrates were found to lead to a substantial increase in the secretion of several growth factors (SDF-1a, BDNF, and bNGF) and result in increased hematopoietic stem cell proliferation.^68^

#### 3.1.2 Ligand Functionalization

The inclusion of ligands to biomaterials was first explored as an approach to promote attachment, but incorporated ligands also have the potential to bias the MSC secretome.^3,5,25,61^ For example, adding GFOGER, a collagen I mimic that can specifically bind to integrin α5β1, led to increased MSC secretion of cytokines IL-8 and IL-6 that are responsible for osteoprogenitor differentiation and controlled levels of osteoclast resorption.^69−71^ The delivery of MSCs loaded in GFOGER functionalized hydrogels led to a substantial increase in bone formation in a murine radial segmental defect as characterized by microcomputed tomography (microCT).^69^

Like stiffness, the surface chemistry of biomaterials can be used to alter the immunomodulatory properties of the MSCs. It was found that both α_V_/α_5_ and α_2_/β_1_ integrins are essential for MSC immunomodulation when cultured on fibrin and collagen hydrogels, respectively.^72^

#### 3.1.3 Geometry/Topography

Broadly, increased MSC secretory activity has been shown in both scaffolds and aggregate culture geometries.^5,61^ Tuning of scaffold porosity and substrate surface patterning can direct MSC clustering and increase secretion activities through cell–cell interactions.^5,61^ The myogenic potential of the MSC secretome for myoblast differentiation has been shown to be linked to the porosity of the scaffold. MSCs cultured in macroporous scaffolds and nanoporous hydrogels were found to have distinct secretion profiles that consequently result in differential paracrine effects on myoblasts.^70^ In this study, it was found that the N-cadherin mediated cell–cell interaction was physically blocked in the nanoporous gel and led to a decrease in myogenesis of myoblasts sensing the MSC secretome.^70^

### 3.2 Differentiation Control

For other clinical applications, cell potency can be increased through differentiation of stem cells into a more mature and specialized cell type. Although many therapeutic considerations for MSCs involve directing their differentiation,^73^ the differentiation of iPSCs, another promising cell type for cell therapies, will be the focus of this section. Inducing pluripotency strategically allows for downstream biomaterial directed differentiation into somatic cells for cell transplantation specific to diseases ranging from neurological to cardiovascular.^73,74^

iPSCs are used as precursors to manufacture both progenitor and differentiated somatic cells in many ongoing clinical trials.^3,75−77^ A particular promise of iPSCs is their ability to serve as a foundational building block that can continuously expand and then be subsequently differentiated to address the disease of interest.^77^ Unfortunately, iPSC-based therapies developed with traditional directed differentiation protocols comprised of soluble factors are still limited by unstable lineage stabilization, heterogeneous phenotype presentation, and scalability concerns.^76^ However, similar to secretome modulation, directed differentiation can be controlled through both biochemical and biomechanical cues and therefore invites the use of engineered biomaterials to address these limitations.

#### 3.2.1 Stiffness/Elasticity

Substrate stiffness can influence the iPSC cell fate. In studies where iPSCs were cultured on poly(vinyl alcohol-*co*-itaconic acid) (P-IA) hydrogels at stiffnesses greater than 30 kPa, iPSCs were shown to undergo long-term expansion while preserving pluripotency.^75^ However, lowering the substrate stiffness to match the stiffness of muscle (1–10 kPa) or the brain and lung (<1 kPa) can direct the differentiation of iPSCs.^75,76^ As such, control of the mechanical environment of iPSCs may provide an inexpensive and easy method to switch between expansion and the directed cell fate.

#### 3.2.2 Ligand Functionalization

Patterned physiochemical cues provide another biomaterial approach to the direct differentiation of iPSCs. More specifically, substrates with tethered inductive moieties have been shown to facilitate neurogenesis, myofibrillogenesis, osteogenesis, and hepatogenesis of iPSCs.^76^ For example, the surface modification of an alginate and poly(c-glutamic acid) composite scaffold with transactivator of transcription (TAT)-VHL peptides, a peptide known to stimulate neurite outgrowth, directly stimulated seeded iPSCs and facilitated neurogenesis.^78^

#### 3.2.3 Geometry/Topography

Naturally occurring ECM nanotopography influences the local migration, polarization, and other functions of surrounding cells and as such can be used to drive similar processes in vitro.^76,79^ For example, polydimethylsiloxane (PDMS) substrates with nanoscale channels (350 nm width) were shown to elevate the expression of neuronal markers.^80^ The observed increase in neuronal differentiation was, in part, shown to be an effect of the topographical features influencing the contact guidance and alignment of seeded iPSCs.^80^

### 3.3 Gene Modification

Genetic editing of cell-based therapies involves the delivery of genetic material such as encoding DNA, mRNA, miRNA, small interfering RNA (siRNA), or small hairpin RNA (shRNA) through either viral or nonviral vectors.^3,81^ Viral vector limitations involve immunogenicity, mutagenesis, and batch variation; however, they benefit from high transfection efficiency and long-term gene expression.^81^ In contrast to viral gene vectors, nonviral gene vectors can avoid integration into the host genome after transfection but suffer from lower transfection efficiency and a shorter gene expression duration.^81^

While efforts for engineering cell potency (improving secretion activities or differentiation) through gene modification are still advancing, we only briefly introduce the approach here to highlight that it is an alternative to direct cell programming. For example, genetically modifying MSCs to yield a conditionally active AKT MSC variant was illustrated to substantially increase vascular endothelial growth factor (VEGF) secretion and highlights the potential of genetic engineering of the secretome.^56,82^ Furthermore, several studies have shown that lentivirus encoding pro-neuronal and subtype-specific transcription factors can drive human fibroblasts into a multipotent transient state that can eventually differentiate into induced neurons.^83,84^ Strategic biomaterial design through the use of inorganic materials, lipid/lipid-like materials, and polymeric materials has been a major focus to improve the transfection efficiency of nonviral gene vectors.^81^

### 3.4 Opportunities and Challenges

Although there have been advances in using biomaterials to engineer cell specificity, there is still a need to better control phenotype to obtain optimally potent cells. Broadly, biomaterials require further optimization to encourage the secretion of specific factors and the suppression of others or to promote the secretion of transgene-encoded proteins. Additionally, biomaterials need further tuning to increase differentiation rates and specificity as well as the efficiency of vector uptake.

To address these remaining challenges, the incorporation of materials that allow for independent control of matrix stiffness and viscoelasticity into cell manufacturing platforms should be addressed. For example, Adu-Berchie et al. have developed a collagen-based ECM mimic with independently tunable mechanical properties (slow relaxing noncovalent collagen interactions and fast relaxing covalent bonds made from a click reaction between norbornene and tetrazine moieties) to generate functionally distinct T-cell populations.^85^ However, incorporation into a cell manufacturing platform, and more specifically a microcarrier system, has yet to be studied.

The translation of ligand density effects on cell phenotypes to the cell manufacturing context is limited. Work has been done to directly study the effect of adhesive ligand density on gene expression of MSCs and yielded in the discovery of a highly specific MSC phenotype that can promote hematopoietic stem cell differentiation through cytokine secretion when cultured on alginate modified gels with a 1500 μM GGGRGDSP peptide density compared to 150 μM.^86^ Moreover, this study also explored how transcriptional programs were affected by combinations of ligand density, material stiffness, and stress–relaxation through RNA-seq.^86^ This methodology is a promising framework that can elucidate how to engineer distinct biophysical features into cells.

With regard to topography/geometry control of cell phenotype, there are still limitations pertaining to the integration of discoveries from microphysiological system assembly. Microphysiological systems recapitulate human physiology to recreate key biological processes and heavily rely on modeling exact tissue architectures.^87^ For example, iPSC-derived neural progenitor cells can differentiate to express more synapse promoting proteins such as synaptophysin based on a surface patterned groove.^88−90^ Integration of surface patterns into microcarriers can lead to improved directed differentiation of phenotype expression.

While general trends between material properties and cell phenotype have been identified, highly specific phenotypic states could benefit from combinatorial design or be obtained from stepwise phenotype shifts. Biomaterial arrays and smart materials that can transition between different material states have the potential to help direct optimized cell potency. Furthermore, better predictive models of how stiffness, ligand density, and topography collectively interact to change cell phenotype can be an outcome of combinatorial studies and shorten the timeline for biomaterial design.^86^

## 4 Biomaterials for the Selection of Cells Post Culture – Decreased Heterogeneity

To harness the full potential of cell-based therapeutics, quality attributes such as cell identity, purity, and potency should be rigorously controlled throughout the biomanufacturing process. However, in many cases, it may be impossible to achieve full homogeneity of the product without some type of postexpansion selection and filtering. Currently many high-throughput devices have been developed that can separate cells based on size and deformability.^91,92^ However, if the goal is to isolate cells based on the expression of characteristic surface markers, new separation approaches need to be further developed.

One such approach would be the use of an affinity-based separations technique that can filter cells based on surface markers characteristic of the desirable cell phenotype.^93^ Cell-binding ligands have been immobilized onto solid substrates, polymer carriers, as well as magnetic particles (MACS) or fluorescent markers (FACS) that can be separated by an electromagnetic field.^93^ More recently, affinity ligands have been displayed on the channels of microfluidic devices.^93,94^

Although iPSC sorting based on the surface markers can increase homogeneity and potency, studies have shown that there is not a correlation between surface markers and potency for MSCs.^95−97^ As such, isolation of potent MSC subtypes is a major limitation. Material advances, such as polymers that change conformation in the presence of a particular secreted protein, might capture desirable MSC phenotypes. However, single cell sensing in a high-throughput manner remains a major technical challenge.

Currently, cell separation techniques post scale-up are not often implemented, and instead, cells classified as minimally or nonpotent are checked for potential deleterious effects. Even if these cells are viewed as therapeutically inert, their inclusion in therapies can reduce the number of potent cells per dose, thus affecting clinical efficacy. The development of cell sorting technologies based on direct potency metrics, therefore, should be a major focus of the field.

## 5 Conclusions and Future Directions

To date, there have been numerous biomaterial interventions that can increase cell potency, scale-up therapeutic cell manufacturing, and reduce cell heterogeneity (Table 1). However, the integration of all of these principles into a single manufacturing pipeline has remained a challenge. Furthermore, the reproducibility and predictability of cell-based therapies have severely impacted clinical translation. In order to advance reproducibility, both heterogeneity within the initial cell population and heterogeneity that compounds during processing steps should be addressed with more sophisticated cell isolation techniques.

**Table 1:**
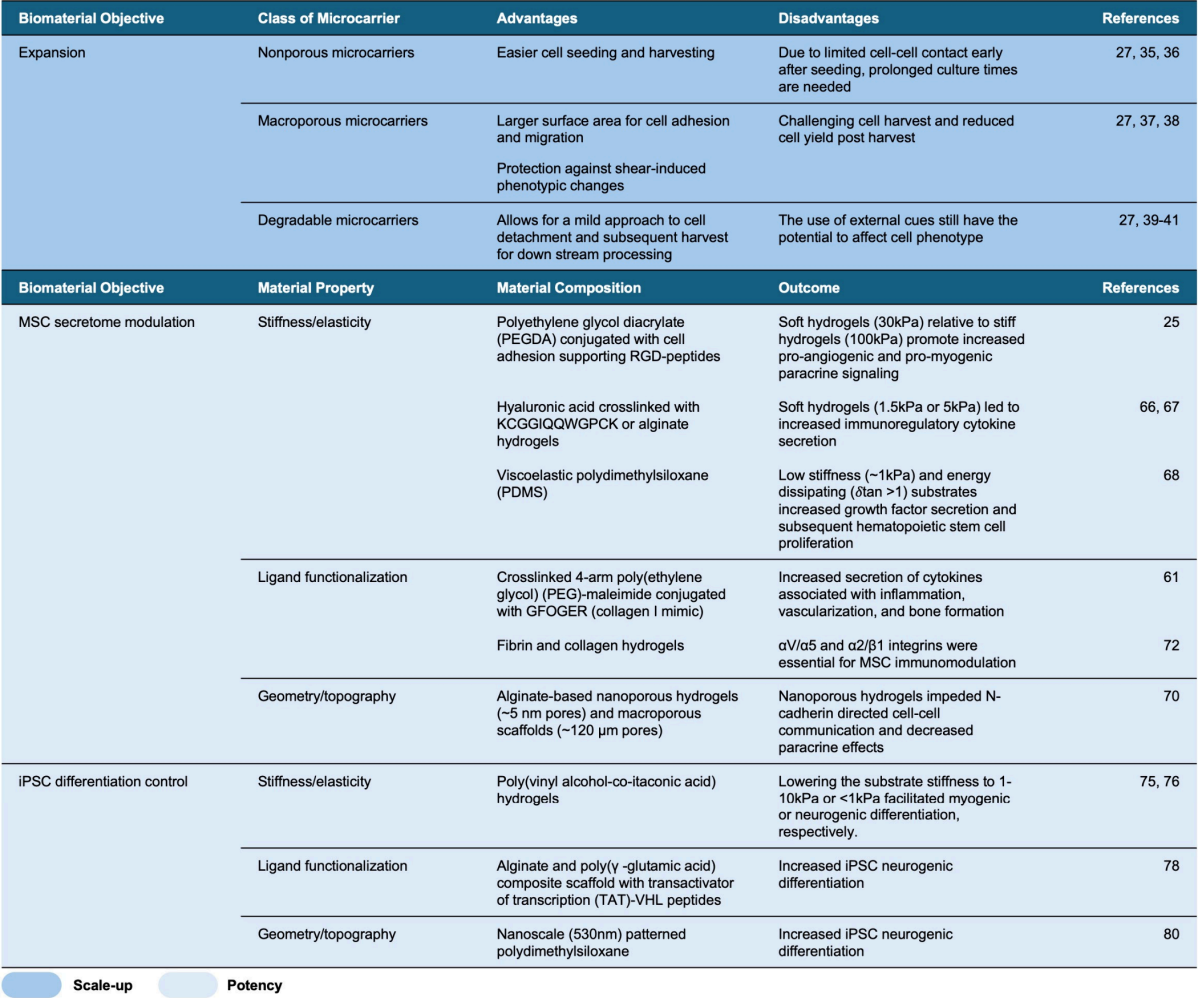
Biomaterials for the Manufacturing of Adherent Cells

The integration of biomaterial cell potency control and scale-up via microcarriers provides a great opportunity to minimize costs and yield more reproducible and potent cells. However, as mentioned above, there can be trade-offs between biomaterial-induced cell expansion and biomaterial-induced potency control. Current integration of these techniques would require sequential operation, where a stiffer microcarrier could be used for increasing cell proliferation and a subsequent culture on softer hydrogel microcarriers could be used for differentiation or secretome control. As an effort to bridge these processing steps, the incorporation of stimuli-responsive hydrogel materials that can adjust their mechanical properties in response to an introduced stimuli could be a promising approach aimed to maximize expansion and potency control within a single bioreactor system in a continuous manner.^98−102^

Even with the many advances in cell potency control, one of the main limitations of translation is the mismatch between theoretical and perceived clinical potency. Inconsistent clinical potency can arise from many factors such as an immunogenic response and clearance of the delivered cells or fast phenotypic shifts in the delivered cells due to the extracellular environment at the site of transplantation.^11,103^ Poor potency can be linked to uncharacterized modes of action and a lack of predictability. As such, a concerted effort to integrate biomaterial recapitulation of the delivery site as a method of screening for in vivo efficacy can be used as a feedback/optimization tool to better engineer highly potency cells. Moreover, selecting for phenotypes that require large activation transition states to enter into a new phenotype may allow for maintained and predictable potency post transplantation.

Finally, biomaterial advances will need to be interfaced with sensors and/or imaging platforms that can detect subcellular markers for desired phenotypes. As a potential first step, noninvasive imaging could be integrated with culture platforms to characterize and/or sort cells based on morphology changes correlated with unique cell phenotypes.^104,105^ While new biomaterials technologies are being developed to screen and filter cells as a method of quality control, essential for clinical translation, limitations exist with high-throughput operation and single cell sensing, and both should be a major consideration with regard to future development of these technologies.

Overall, the use of biomaterials in cell manufacturing provides an innovative and exciting means to directly interface with and thus control cell behavior, with the eventual goal of manufacturing highly potent cell therapies that can treat a wide range of diseases.
